# Size and dielectric properties of skeletal stem cells change critically after enrichment and expansion from human bone marrow: consequences for microfluidic cell sorting

**DOI:** 10.1098/rsif.2017.0233

**Published:** 2017-08-23

**Authors:** Miguel Xavier, María C. de Andrés, Daniel Spencer, Richard O. C. Oreffo, Hywel Morgan

**Affiliations:** 1 Faculty of Physical Sciences and Engineering, and Institute for Life Sciences, University of Southampton, Southampton SO17 1BJ, UK; 2 Centre for Human Development, Stem Cells and Regeneration, Institute of Developmental Sciences, Southampton General Hospital, Tremona Road, SO16 6YD Southampton, UK

**Keywords:** skeletal stem cells, cell sorting, impedance cytometry, label-free, microfluidics, tissue regeneration

## Abstract

The capacity of bone and cartilage to regenerate can be attributed to skeletal stem cells (SSCs) that reside within the bone marrow (BM). Given SSCs are rare and lack specific surface markers, antibody-based sorting has failed to deliver the cell purity required for clinical translation. Microfluidics offers new methods of isolating cells based on biophysical features including, but not limited to, size, electrical properties and stiffness. Here we report the characterization of the dielectric properties of unexpanded SSCs using single-cell microfluidic impedance cytometry (MIC). Unexpanded SSCs had a mean size of 9.0 µm; larger than the majority of BM cells. During expansion, often used to purify and increase the number of SSCs, cell size and membrane capacitance increased significantly, highlighting the importance of characterizing unaltered SSCs. In addition, MIC was used to track the osteogenic differentiation of SSCs and showed an increased membrane capacitance with differentiation. The electrical properties of primary SSCs were indistinct from other BM cells precluding its use as an isolation method. However, the current studies indicate that cell size in combination with another biophysical parameter, such as stiffness, could be used to design label-free devices for sorting SSCs with significant clinical impact.

## 1. Introduction

Skeletal stem cells (SSCs) are present in bone marrow (BM) and have the capacity to form fat, cartilage and bone. Their differentiation capacity is restricted to skeletal lineages and although the terms SSCs and mesenchymal stem cells (MSCs) have been used interchangeably, SSCs should be distinguished from MSCs which are reported to exist in extra-skeletal tissues and do not contribute to skeletal development [1–3].

In an increasingly aged society, the potential to harness stem cells to promote tissue regeneration using stem cell therapies or tissue-engineering approaches is of paramount importance. However, at present there are no protocols for the isolation of a homogeneous SSC population from human BM [3]. Consequently, despite their potential both in clinical applications [4,5] and for R&D, SSCs have only been used as a heterogeneous mixture of human bone marrow mononuclear cells (hBMMNCs) that include stem cells, other stromal progenitors and cells of the haematopoietic lineage.

The frequency of SSCs in the BM is extremely low, estimated at fewer than 1 in 10 000 hBMMNCs [6]. Current antibody-based enrichment protocols target multiple cell surface markers but, in the absence of a defined and selective marker for the SSCs, cannot provide a homogeneous stem cell population. One strategy employed a combinatorial approach targeting CD146 (melanoma cell adhesion molecule) [7–9], together with the Stro-1 antibody [10]. Sorting was achieved using both magnetic and fluorescence-activated cell sorting, increasing the number of colony forming units-fibroblastic (CFU-F) up to 2000-fold [11]. The Stro-1 antibody binds to an antigen recently identified as Heat Shock Cognate 70 (HSC70/HSPA8) [12], on the surface of approximately 10% of hBMMNCs and has been used for nearly three decades to enrich for adherent, high growth-potential CFU-F [10,13]. CD146 exists on a marginal sub-population of hBMMNCs that contribute to the regulation of haematopoiesis—a key feature of SSCs [1,8,9,14]. However, neither of these markers is specific to SSCs limiting the efficiency of antibody-dependent isolation methods.

A crucial step in most isolation protocols to enrich for the skeletal stem and progenitor population is the isolation of cells that adhere to tissue culture plastic, as this depletes the non-adherent populations. However, it is not clear whether plastic adherence alters the SSC phenotype from its original state in the BM microenvironment. Following selection by adherence, SSCs are commonly expanded for several weeks before assay, which can further alter their properties. Indeed, there is emerging evidence that human BM stromal cells become (i) larger [15,16], (ii) stiffer [17], (iii) less proliferative [18–21], (iv) display increased heterogeneity [16,21], and (v) display loss of surface markers [10,22–24] and multipotent differentiation potential [19–21] with passage.

Microfluidics offers new approaches to cell sorting that can isolate rare cells based solely on the cells biophysical parameters. For a recent review in the context of SSC isolation, see [25]. Crucially, these methods do not use antibodies and are thus ‘label-free’. Typical biophysical parameters include size, stiffness, shape and acoustic and dielectric properties. The design of such microfluidic devices requires *a priori* knowledge of the unique biophysical features that discriminate SSCs from other cells found in the BM. To date, a dominant body of work within the skeletal cell arena has employed passaged cells limiting relevance to SSCs. Therefore, it is important to study SSCs in their original state—before plastic adherence and expansion.

One commonly used label-free sorting method is dielectrophoresis (DEP), which has been widely used to sort cells based on a combination of size and dielectric properties, typically membrane capacitance [26,27]. For example, it has been used to isolate circulating tumour cells from peripheral blood of cancer patients as these cells are typically larger and have a higher membrane capacitance than healthy leucocytes [28]. DEP has also been used to sort stem cells either from their progenies [29–31] or from their tissue of origin [32–34] but with moderate success. In one example, putative adipose tissue-derived stem cells were enriched from digested adipose tissue by 14-fold, but mainly due to the removal of cell debris and erythrocytes, as the positive fraction was still largely contaminated (73%) with CD45^+^ nucleated cells [32].

DEP is widely used to measure the dielectric properties of a population of cells by analysing their response to an electric field with varying frequencies [26,35–37]. Flanagan *et al.* [38] showed that mouse neural stem and precursor cell (NSPC) mixtures have different dielectric properties from neurons and astrocytes. The same authors later showed that NSPCs displayed different DEP responses depending on the population bias towards astrogenic or neurogenic differentiation in both human [39] and mouse [31] cells. Also using DEP, human embryonic stem cell lines were shown to undergo a significant increase in membrane capacitance following differentiation into an MSC-like phenotype [37]. We used DEP to characterize the dielectric properties of routinely expanded SSCs and of MG-63 and Saos-2 cell lines, representative of early and mature bone cell populations, respectively [40].

Microfluidic impedance cytometry (MIC) is a non-invasive, high-throughput single-cell characterization technique that measures the size and dielectric properties of cells in flow [41]. High throughput is particularly valuable as it allows studying rare cell populations such as SSCs in BM. MIC was recently used to study the differentiation of rat neural stem cells [42] and mouse embryonic stem cells (mESCs) [43,44]. The differentiation process of mESCs was associated with an increase in the cells membrane capacitance indicating the potential of MIC to be used to monitor stem cell differentiation.

In this work, we have used MIC to characterize the size and dielectric properties of primary human SSCs derived from unexpanded human BM samples. SSCs were pre-enriched using Stro-1^+^ magnetic isolation (MACS), and progenitor and SSC populations within the hBMMNCs sub-population were further identified with CD146^+^ fluorescent detection. The size and membrane capacitance of SSCs was compared with other hBMMNCs, and analysed as a function of cell expansion and passage. We also investigated changes in cell proliferation, alkaline phosphatase (ALP) activity and the expression of relevant genes of interest. In addition, the dielectric properties of SSCs were measured following osteogenic differentiation. With this study, we aim to emphasize the importance of using unexpanded SSC cultures and to generate critical information on the biophysical properties of SSCs in the human BM that will allow their label-free sorting with significant clinical impact.

## 2. Material and methods

### 2.1. Cell culture

#### 2.1.1. Isolation and expansion of primary human SSCs

Human BM samples were obtained from patients undergoing total hip replacement surgeries at the Spire Southampton Hospital, with full patient consent. Only tissue that would have been discarded was used, with approval of the Southampton and South West Hampshire Research Ethics Committee (Ref no. 194/ 99/1 and 210/01). Following cell extraction from the BM, samples were washed with plain α-MEM and the cell suspension was filtered through a 70 µm cell strainer and layered upon Lymphoprep™ to remove red blood cells and the majority of granulocytes by density centrifugation. The BMMNC fraction was collected from the ‘buffy coat’ and incubated with the Stro-1 monoclonal antibody (IgM) from mouse hybridoma produced *in loco*. The SSC-enriched Stro-1^+^ cell population was isolated by magnetic separation of cells labelled with anti-mouse IgM microbeads, as previously detailed [45,46].

**Figure 1.**
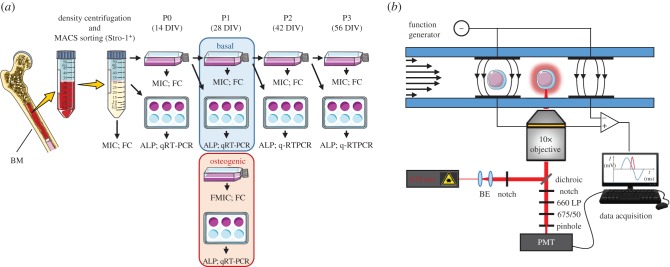
(*a*) Schematic diagram of the experimental protocol. Human bone marrow mononuclear cells (hBMMNCs) were obtained by density centrifugation of BM samples from patients undergoing total hip replacement surgery and enriched by MACS sorting of the Stro-1^+^ population. Before expansion and at each passage and corresponding day *in vitro* (DIV), cells were analysed using microfluidic impedance cytometry (MIC), flow cytometry (FC), alkaline phosphatase (ALP) activity and/or qRT-PCR. At passage 1, the same analyses were performed to detect changes in cells following osteogenic differentiation. (*b*) The impedance cytometry set-up including the confocal-optical detection. Cells flow through the microchannel, passing between pairs of electrodes and the optical detection region. The fluorescence properties of the cell were measured simultaneously with the impedance allowing direct correlation of the electrical and fluorescent properties on a single-cell basis.

The enriched SSC population was plated at a cell density of 1 × 10^4^ cells cm^−2^ in α-MEM supplemented with 10% fetal calf serum (FCS) (v/v), 100 U ml^−1^ penicillin and 100 µg ml^−1^ streptomycin (basal medium) and maintained in a humidified chamber at 37°C and 5% CO_2_ (passage 0). After one week of culture, non-adherent cells were washed away, the medium was replenished and subsequently changed every other day. For passage, cells were pre-treated with collagenase IV (200 µg ml^−1^) in plain α-MEM for 40 min and lifted using 0.025% (w/v) Trypsin–EDTA with 0.05% glucose for 10 min at 37°C. Cells were re-plated at a density of 2 × 10^2^ cells cm^−2^. A diagram of this procedure with time-points of analysis is shown in figure 1*a*.

##### 2.1.1.1. Osteogenic differentiation

After expansion for 14 days in basal conditions, SSCs were passaged at a cell seeding density of 2 × 10^2^ cells cm^−2^ and cultured in osteogenic medium consisting of basal medium supplemented with dexamethasone (10 nM) and ascorbate-2-phosphate (100 µM). The medium was replenished every other day and the cells were analysed after 14 days [47].

#### 2.1.2. MG-63 and Saos-2

MG-63 human osteosarcoma cells, from passages 27 to 30, and Saos-2 human osteosarcoma cells, from passages 26 to 28, were cultured in DMEM and α-MEM, respectively, supplemented with 10% FCS (v/v), 100 U ml^−1^ penicillin and 100 µg ml^−1^ streptomycin, and maintained in a humidified chamber at 37°C and 5% CO_2_. The culture medium was replenished every 2–3 days and the cells were routinely sub-cultured assuring a maximum confluence of 70%, being detached using 0.025% (w/v) Trypsin–EDTA with 0.05% glucose for 5 min at 37°C and re-plated at a cell seeding density of 2–4 × 10^4^ cells cm^−2^.

#### 2.1.3. HL-60

HL-60, human peripheral blood promyelocytic leukaemia cells, were cultured in RPMI-1640 supplemented with 10% FCS (v/v), 100 U ml^−1^ penicillin and 100 µg ml^−1^ streptomycin, and maintained in a humidified chamber at 37°C and 5% CO_2_. The cells were split every other day to provide a final concentration of 2 × 10^5^ cells ml^−1^.

### 2.2. Fluorescent antibody labelling and sample preparation

Human BMMNCs in suspension were incubated with blocking buffer (PBS 1×, 10% AB Human serum (v/v), 5% FCS (v/v) and 1% (w/v) BSA) to minimize non-specific antibody bonding. After blocking, cells were incubated in Alexa Fluor^®^ 647-conjugated mouse anti-human CD146 antibody (563619 BD Pharmingen™) for 30 min at 4°C under agitation. The cells were then incubated with Stro-1 antibody from a mouse hybridoma followed by incubation with Alexa Fluor^®^ 488-conjugated goat anti-mouse IgM antibody (A-21042 from Invitrogen™). Non-specific binding was controlled using Alexa Fluor^®^ 647-conjugated Mouse IgG1 κ Isotype Control (557714 BD Pharmingen^TM^) and an IgM Isotype Control from a murine myeloma (M5909 SIGMA).

### 2.3. Fluorescence cytometry

Labelled hBMMNCs were suspended in running buffer (PBS 1×, 2 mM EDTA and 0.5% (w/v) BSA), filtered through a 100 µm cell strainer and analysed using a BD^®^ Accuri C6 flow cytometer (Becton, Dickinson and Company, Franklin Lakes, NJ, USA). Data analyses were performed using FlowJo V10 software (FlowJo LLC, Ashland, OR, USA). Single cells were gated for linearity in the forward scatter (FSC-H versus FSC-A) plot and typically, 25 000 events were acquired for each measurement inside the single-cell gate. Events were considered fluorescently positive above the threshold at which less than 1% of the cells were positive for the matched isotype control.

### 2.4. Fluorescence-assisted microfluidic impedance cytometry

#### 2.4.1. Microfluidic chip and impedance cytometer set-up

Figure 1*b* shows a diagram of the single-cell analysis system. The microfluidic chip is fabricated from glass with a microfluidic channel (30 × 40 µm), defined in SU8 photoresist, through which cells flow. A detailed fabrication protocol of the MIC chips can be found elsewhere [48]. Platinum micro-electrodes were lithographically patterned onto the glass and connected to AC sinusoidal voltages (4Vpp) at fixed frequencies. When a particle moves between the electrode pairs, a differential current flows in the system. One pair of electrodes measures the electrical signal from the particle while the other pair acts as reference. Single-cell impedance was measured using a custom trans-impedance amplifier and an impedance spectroscope (HF2IS, Zurich Instruments AG, Zurich, Switzerland). Simultaneously, when applicable cells were illuminated by a focused 100 mW 635 nm laser beam (LRD-0635-PF, Laserglow Technologies, Toronto, ON, Canada) exciting fluorescence from fluorescently labelled CD146^+^ cells. The fluorescence emission was captured by the objective lens (10×, N.A. 0.30), passed through dichroic and band pass filter sets, spatially filtered using a pinhole and could be detected in the wavelength window from 660 to 700 nm using a photomultiplier (Hamamatsu Photonics, Hamamatsu, Japan) [49]. The simultaneous collection of both fluorescence and impedance allows independent identification and verification of cell phenotype. The data were processed and analysed using custom software written in Matlab™ (Mathworks, Inc. Natick, MA, USA) [50]. The impedance data were used to trigger the data acquisition and a peak detection algorithm was used to detect the fluorescence signals.

#### 2.4.2. Sample preparation for MIC

The MIC chips were cleaned with 1 M NaOH and dH_2_O, and primed with running buffer (PBS 1x, 2 mM EDTA and 0.5% (w/v) BSA) before each experiment. Cells were suspended in running buffer at a concentration of 2–2.5 × 10^5^ cells ml^−1^ and mixed with 7 μm polystyrene (PS) beads (in PBS, 0.01% Tween-20) at 0.5–1 × 10^5^ particles ml^−1^. PS beads have constant dielectric properties and were used to normalize the MIC results. The cell/bead suspension was pumped through the impedance cytometer at 40 µl min^−1^.

### 2.5. Alkaline phosphatase staining

Stro-1-enriched SSCs cultured in six-well plates were washed with PBS and fixed with 95% ethanol in dH_2_O for 10–15 min. Fixed cultures were incubated with a Fast Violet B salt (0.24 mg ml^−1^) and Naphtol AS-MX solution (4% v/v) in dH_2_O for 40 min at 37°C in a humidified atmosphere protected from light. Cultures were washed with dH_2_O and imaged using a dissecting stereoscope [7].

### 2.6. ALP activity and DNA quantification

Stro-1-enriched SSCs cultured in six-well plates were washed with PBS and fixed with 95% ethanol in dH_2_O for 10–15 min. Cells were lysed using 0.05% (v/v) Triton™ X-100 solution in dH_2_O followed by three freeze–thaw cycles. Total DNA was quantified against a standard curve (ssDNA from salmon testes) by addition of Quant-iT™ PicoGreen^®^ dsDNA diluted in TE buffer and measurement of the emitted fluorescence (*λ*_ex_ = 480 nm, *λ*_em_ = 520 nm) using a FLx800™ fluorescence reader (BioTek, Winooski, VT, US). ALP activity was quantified against a standard curve (p-nitrophenol) by incubation of the cell lysate with a phosphatase substrate solution (1.33 mg ml^−1^ *p*-nitrophenyl phosphate in 0.5 M alkaline buffer solution) at 37°C in the dark, under gentle agitation. The reaction was terminated by addition of 1 M NaOH in dH_2_O after 40 min and absorbance was measured at 410 nm using an ELx800™ absorbance reader (BioTek) [22,45].

### 2.7. Gene expression analysis

#### 2.7.1. RNA extraction and complementary DNA synthesis

Total RNA was extracted from cultured cells using an RNeasy Mini Kit (Qiagen, Hilden, Germany). RNA concentration was measured using a NanoDrop 1000 spectrophotometer (Thermo Fisher Scientific, Waltham, MA, USA) and the RNA was reverse-transcribed using TaqMan Reverse-Transcription Reagents (Applied Biosystems, Foster City, CA, USA).

#### 2.7.2. Quantitative reverse-transcription polymerase chain reaction

Relative quantification of gene expression was performed as reported previously [51] using an ABI Prism 7500 detection system (Applied Biosystems). Primer Express 3.0 software (Applied Biosystems) was used to design the primers, which are shown in table 1. A 20 µl reaction mixture was prepared in duplicate, containing 1 µl of complementary DNA, 10 µl of GoTaq qPCR Master Mix (Promega, Madison, WI, USA) and 1 µM of each primer. Thermal cycler conditions included an initial activation step at 95°C for 10 min, followed by a two-step PCR programme of 95°C for 15 s and 60°C for 60 s for 40 cycles. The 2^−ΔΔCt^ method was used for relative quantification of gene expression, and the data were normalized to the expression of β-actin and compared to the expression levels of each gene at passage 0 or basal conditions, accordingly.

**Table 1.** Primers used for qRT-PCR (F: forward and R: reverse).

transcript	abbreviation (name, length)	primer sequence (5′–3′)
beta-actin	*ACTB* (82)	F: GGCATCCTCACCCTGAAGTA R: AGGTGTGGTGCCAGATTTTC
alkaline phosphatase (ALP)	*ALPL* (86)	F: GGAACTCCTGACCCTTGACC R: TCCTGTTCAGCTCGTACTGC
runt-related transcription factor 2	*RUNX2* (78)	F: GTAGATGGACCTCGGGAACC R: GAGGCGGTCAGAGAACAAAC
osteocalcin	*BGLAP* (110)	F: AAGAGACCCAGGCGCTACCT R: AACTCGTCACAGTCCGGATTG
transcription Factor SOX2	*SOX2* (95)	F: CAAGATGCACAACTCGGAGA R: GCTTAGCCTCGTCGATGAAC
alpha-1 Type I collagen	*COL1A1* (52)	F: GAGTGCTGTCCCGTCTGC R: TTTCTTGGTCGGTGGGTG
alpha-1 Type II collagen	*COL2A1* (58)	F: CCTGGTCCCCCTGGTCTTGG R: CATCAAATCCTCCAGCCATC
nucleostemin	*GNL3* (98)	F: GGGAAGATAACCAAGCGTGTG R: CCTCCAAGAAGTTTCCAAAGG
peroxisome proliferator activated receptor gamma	*PPARG* (108)	F: GGGCGATCTTGACAGGAAAG R: GGGGGGTGATGTGTTTGAACTTG
fatty acid binding protein 4	*FABP4* (108)	F: TAGATGGGGGTGTCCTGGTA R: CGCATTCCACCACCAGTT
transcription factor SOX9	*SOX9* (74)	F: CCCTTCAACCTCCCACACTA R: TGGTGGTCGGTGTAGTCGTA

### 2.8. Statistical analysis

All results were obtained from at least four independent measurements from different patients. Results are represented as mean ± s.d. unless stated otherwise, with graphs prepared using GraphPad Prism v.7 (San Diego, CA, USA). Statistical analysis was performed using the IBM SPSS software package v. 21 (IBM, NY, USA). Data distributions were tested for normality using the Shapiro–Wilk test and statistical significance was tested using one-way analysis of variance with Tukey's post hoc test for samples following a normal distribution or the Mann–Whitney *U*-test for samples not following a normal distribution. The statistical significance of the differences in gene expression between basal and osteogenic conditions was assessed using Student's *t*-test.

### 2.9. Materials

BioWhittaker^®^ Dulbecco's modified Eagle medium with glucose and l-glutamine (DMEM), Alpha minimum essential medium with desoxyribonucleotides, ribonucleotides and ultra-glutamine (α-MEM), RPMI-1640, Dulbecco's phosphate buffered saline, FCS and trypsin/EDTA with glucose were obtained from Lonza (Basel, Switzerland). Penicillin–streptomycin 100×, AB human serum, collagenase IV, ethylenediamine tetra-acetic acid (EDTA), Naphtol AS-MX, Fast Violet Salt B, Triton™ X-100, ssDNA from Salmon testes, Igepal^®^ CA-630, alkaline buffer solution 1.5 M, phosphatase substrate, 4-nitrophenol, 7 μm polystyrene beads and Tween^®^ 20 were purchased from Sigma-Aldrich (St. Louis, MO, USA). Quant-iT™ PicoGreen^®^ dsDNA Reagent was from Thermo Fisher Scientific (Waltham, MA, USA). Bovine serum albumin (BSA) was obtained from GE Healthcare (Chicago, IL, USA). Lymphoprep™ was bought from Stem Cell Technologies (Vancouver, Canada). Anti-mouse IgM microbeads, LS MACS™ columns and the QuadroMACS™ separator were purchased from Miltenyi Biotec (Bergisch Gladbach, Germany). All reagents were used as received and according to the manufacturer's recommendations.

## 3. Results and discussion

### 3.1. Frequency of Stro-1^+^ and CD146^+^ cells following Stro-1 enrichment and cell expansion

**Figure 2.**
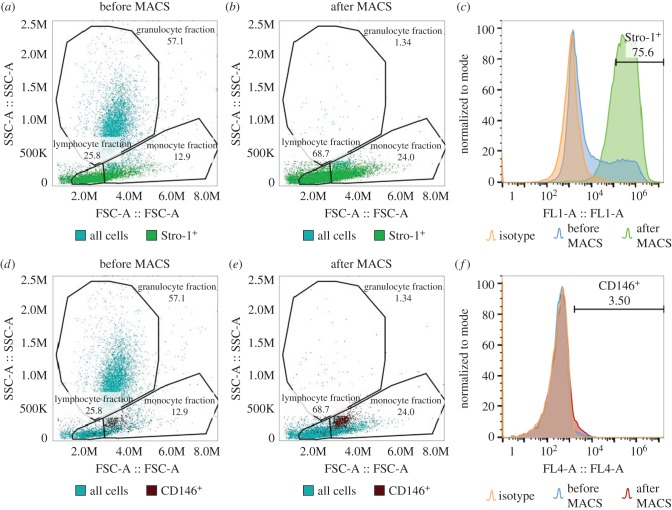
Fluorescence flow cytometry of unexpanded hBMMNCs. (*a*,*b*,*d,e*) Plots (forward versus side scatter) of unexpanded hBMMNCs before (*a*,*d*) and after (*b*,*e*) enrichment of Stro-1^+^ cells by magnetic-activated cell sorting (MACS). The three gates show the fraction of cells falling inside typical FSC and SSC distributions for lymphocytes, granulocytes and monocytes as previously shown [52]. (*c,f*) Frequency histograms of the fluorescence intensity of hBMMNCs before and after enrichment by MACS and the respective matched isotype controls. Images (*a*–*c*) and (*d*–*f*) refer to the expression levels detected using the Stro-1 antibody and an anti-CD146 antibody, respectively.

**Figure 3.**
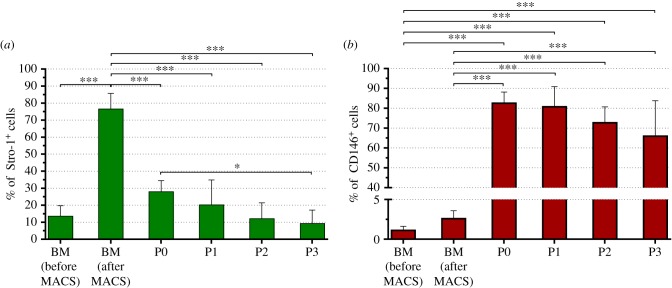
Fluorescence flow cytometry of hBMMNCs. Frequency of Stro-1^+^ (*a*) and CD146^+^ (*b*) hBMMNCs in the BM, before and after enrichment of the Stro-1^+^ population by MACS, and following cell expansion under basal culture conditions (passages 0–3). Values represent mean ± s.d. (*N* ≥ 4, *p < 0.05, ***p < 0.001). (Online version in colour.)

The frequency of SSCs in the BM is estimated at fewer than 1 in every 10 000 nucleated hBMMNCs [6]. Consequently, to study SSCs in unexpanded human BM samples these cells must be pre-enriched. Unsorted hBMMNCs were analysed by flow cytometry and a representative scatter plot (FSC-A versus SSC-A) of the sample is presented in figure 2*a*. The populations fall into three gates (lymphocyte, monocyte and granulocyte fractions). The majority of cells present within the granulocyte fraction were depleted after MACS sorting of Stro-1^+^ cells (figure 2*b*) and the largest enrichment was observed within the lymphocyte fraction. This can be attributed to nucleated erythroid progenitor cells, which contribute to more than 95% of the Stro-1^+^ cells in the BM [10], but display typically lower FSC signals [53]. The Stro-1^+^ hBMMNCs were enriched from 13.8 ± 5.9% (*N* = 6) in the BM to 76.7 ± 9.0% (*N* = 6, *p* < 0.001) after MACS (figures 2*a*–*c* and 3*a*). MACS enrichment with Stro-1 increased the frequency of CD146^+^ cells more than twofold from 1.2 ± 0.4% (*N* = 4) in the BM to 2.7 ± 0.9% (*N* = 6) (figures 2*d*–*f* and 3*b*). The vast majority of CD146^+^ cells were localized within the monocyte fraction, as previously reported for putative SSC populations [52]. Enrichment of the Stro-1^+^ hBMMNCs using MACS and restriction of subsequent analyses to the CD146^+^ fraction increased the frequency of SSCs in the samples from 1 in 10 000 to around 1 in every 40 (2.7% CD146^+^ of 13.8% Stro-1^+^) hBMMNCs, a significant sub-population.

The frequency of CD146^+^ and Stro-1^+^ cells was investigated as a function of cell passage. Figure 3*a* shows a marked decrease in the Stro-1^+^ cell fraction from 76.7 ± 9.0% (*N* = 6) in the BM after MACS to 28.3 ± 6.3% (*N* = 5, *p* < 0.001) after 14 days in culture (passage 0). The percentage of Stro-1^+^ cells continued to decrease significantly with passage to just 8.8 ± 8.4% (*N* = 5, *p* < 0.05 versus P0) at passage 3, corresponding to 56 days *in vitro* (DIV). These results are in agreement with previous reports. In [10], the percentage of Stro-1^+^ cells in adult human BM cells declined to 8.5% after six weeks in culture, and in [22], Stro-1^+^ cells were completely absent at passage 7, after 21–35 DIV, in human fetal femur-derived BM cell cultures. Stewart *et al*. [23] reported the percentage of Stro-1^+^-enriched hBMMNCs to be around 21 ± 6% (mean ± s.e.m.) at passage 1 after 28 days in culture which correlates with our findings at 20.4 ± 14.4% (*N* = 5). Conversely, the frequency of CD146^+^ cells increased markedly at passage 0 (82.9 ± 5.3%, *N* = 5) compared with immediately after MACS isolation (2.7 ± 0.9%, *N* = 6, *p* < 0.001). This reflects the efficiency of cell adhesion as part of the current SSC isolation protocol which removes non-adherent cells from the haematopoietic lineage that are Stro-1^+^ but CD146^−^, as is the case for nucleated erythroid progenitor cells [10,53]. With cell expansion and passage, the percentage of CD146^+^ appears to gradually decline, although this trend did not reach statistical significance. A previous study has shown a minimal decrease in the expression of CD146 after six passages [24]. Electronic supplementary material, figure S1 shows individual flow cytometry data for all six patient BM samples.

### 3.2. Microfluidic impedance cytometry measurements of Stro-1^+^-enriched human SSCs unexpanded and following cell expansion: cell size

**Figure 4.**
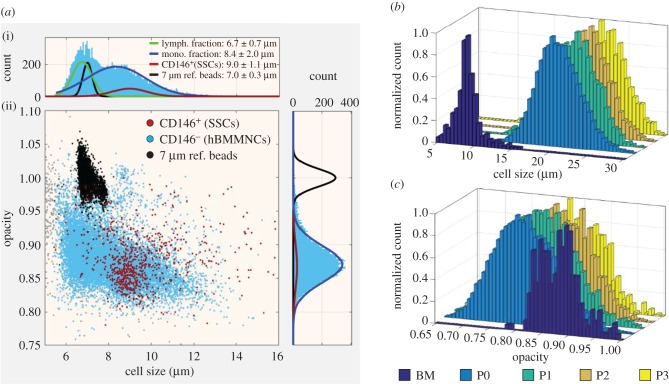
Fluorescence-coupled microfluidic impedance cytometry of Stro-1-enriched hBMMNCs. (*a*) Scatter plot of cell size (µm) versus electrical opacity (normalized to 7 μm diameter reference beads) of Stro-1^+^-enriched hBMMNCs obtained from one individual patient. Histograms (i) and (ii) demonstrate the cell size and opacity distributions, respectively, with colour-coded Gaussian distributions fitted to the data. The dark red line represents the distribution of CD146^+^ cells, representative of skeletal stem cells (SSCs). (*b,c*) Histograms of the distribution of cell size and electrical opacity with increasing cell passage from one individual patient.

Conventional flow cytometry does not provide a precise cell size measurement because the forward light scatter (FSC) signal does not scale linearly with size. Figure 4*a* shows an impedance scatter plot of Stro-1^+^ unexpanded hBMMNCs obtained from one individual patient. For individual impedance scatter plots of all six patient BM samples, see the electronic supplementary material, figure S2. Simultaneous fluorescence detection uniquely identified the CD146^+^ cells (dark red) and impedance was measured simultaneously at two frequencies (500 kHz and 2 MHz). At a low frequency (500 kHz), cells are insulating and for viable cells electrical impedance scales linearly with cell volume. Thus, MIC provides accurate data for cell size. For absolute size determination and to eliminate drift and experiment-to-experiment variability, polymer beads with a known size were mixed with the cells. At 2 MHz, it is possible to detect changes in cells membrane capacitance, which reflects differences in the composition and morphology of the cell plasma membrane (electronic supplementary material, figure S3). However, to consider the influence of cell size differences at 2 MHz, the measurement must be normalized to the cell volume to give the so-called electrical opacity: the impedance ratio at a higher to a lower frequency (2 MHz/500 kHz).

**Figure 5.**
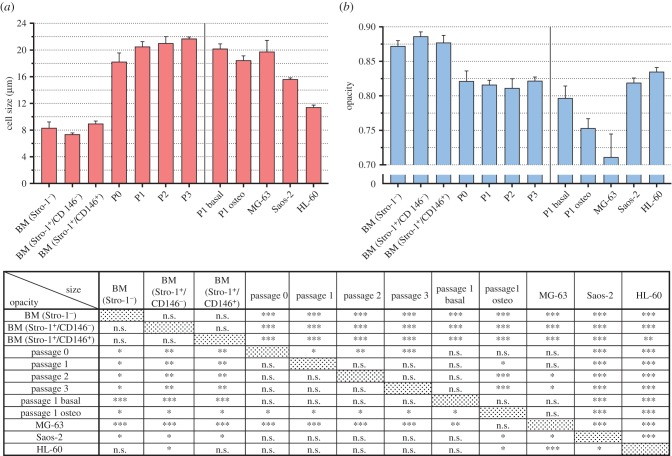
Size and electrical opacity of cells analysed by microfluidic impedance cytometry. Bar charts summarizing the cell size (*a*) and electrical opacity (*b*) of hBMMNCs in the BM, after expansion at passages 0–3, and following osteogenic differentiation (P1 Osteo). Within the BM, cell size and opacity were measured for Stro-1^−^ cells, and both the CD146^−^ and CD146^+^ populations within the Stro-1^+^ fraction. Skeletal stem cells (SSCs) are contained within the Stro-1^+^/CD146^+^ population. Also shown are data for two adherent (MG-63 and Saos-2) and one suspension (HL-60) human cancer cell lines. The appended table summarizes the statistical analyses of the data with the top-right and bottom-left values showing the *p*-values for cell size and opacity, respectively. Values represent mean ± s.d. (*N* ≥ 3, *p < 0.05, **p < 0.01, ***p < 0.001, n.s., not significant). (Online version in colour.)

In figure 4*a*, histograms illustrate the distribution of size and opacity of the cells along with a Gaussian fit for the size distribution of CD146^+^ (dark red line) and CD146^−^ cells (blue and green). Given the comparable size distributions, these can be associated with the lymphocyte and monocyte fractions from figure 2*e*, respectively. The mean cell size and opacity values calculated from a minimum of three independent measurements for each analysed group of cells are presented in figure 5*a,b*, with the independent measurement values for each patient shown in the electronic supplementary material, figure S4.

The size of the CD146^+^ cells in the Stro-1-enriched unexpanded hBMMNCs was observed to overlap significantly with other (CD146^−^) hBMMNCs (figure 4*a*) indicating that cell size is not a unique marker for SSCs and suggesting that these cells could not be isolated based solely on size. The SSCs were found within the largest cell fraction in the BM (figure 5*a*), with a measured diameter of 9.0 ± 0.4 µm (*N* = 6), compared to 7.4 ± 0.2 µm (*N* = 4) and 8.3 ± 0.9 µm (*N* = 4) for Stro-1^+^/CD146^−^ and Stro-1^−^ cells, respectively.

The size of the SSCs was significantly larger following adherence and expansion (figure 4*b*). As early as passage 0 (14 DIV), the average cell size was noted to more than double, increasing to 18.2 ± 1.3 µm (figure 5*a*, *N* = 4, *p* < 0.001). The cell size continued to increase gradually to an average of 21.7 ± 0.3 µm at passage 3, significantly larger than at passage 0 (*N* = 4, *p* < 0.001). The average size range observed for expanded cells (18.2–21.7 µm) is in agreement with the sizes reported for expanded hBMMNCs estimated using microscopy [15,16,40,54] or deformability cytometry [55]. These results also support previous findings that hBMMNCs increase in size with culture expansion [15,16,56], associated with a loss of both self-replicative [16] and differentiation capacity [54,56]. The substantial size difference found between unexpanded and expanded SSCs highlights the phenotypic changes that SSCs undergo during expansion, and emphasize the need to study SSCs in their unaltered state. Indeed, if the size of expanded SSCs was observed in the BM, size-based SSC isolation would become trivial.

Nevertheless, the average cell size found for SSCs in the BM (9.0 ± 0.4 µm) was the largest of the cell populations analysed. Cell size is an important parameter that strongly dictates the outcome of label-free microfluidic cell-sorting approaches [25]. These results indicate that cell size could be used in combination with another biophysical parameter for SSC purification from human BM. Using real-time deformability cytometry, a contactless microfluidic technique for high-throughput mechanical characterization of single cells, we have previously shown that SSCs at passage 0 are significantly stiffer than other cells present in the BM including lymphocytes, monocytes and granulocytes [55]. Combining cell size with higher cell stiffness, could allow separation using techniques that are sensitive to both size and deformation, such as inertial microfluidics [57] or deterministic lateral displacement (DLD) [58]. Given the phenotypic changes reported here for SSCs following adherence and expansion it would be important to determine whether the higher cell stiffness observed for SSCs at passage 0 [55] could be verified in unexpanded samples.

### 3.3. Microfluidic impedance cytometry measurements of Stro-1^+^-enriched human SSCs unexpanded and following cell expansion: membrane capacitance

Figure 4*a* shows that the opacity of CD146^+^ and CD146^−^ cells completely overlap, with no significant differences between the average opacity of Stro-1^−^ (0.87 ± 0.01, *N* = 4), Stro-1^+^/CD146^−^ (0.88 ± 0.01, *N* = 4) and Stro-1^+^/CD146^+^ (0.88 ± 0.01, *N* = 6) cells. These data indicate that opacity cannot be used as a label-free marker for the SSCs contained within the Stro-1^+^/CD146^+^ sub-population. They also show that the membrane capacitance of unexpanded SSCs is indistinct from other cells in the BM indicating that sorting SSCs from human BM using a technique such as DEP is unlikely, in isolation, to yield pure sub-populations.

**Figure 6.**
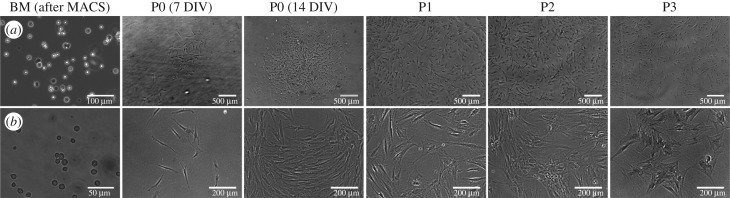
Optical microscopy. Images show apparent changes in the size and morphology of unexpanded hBMMNCs after enrichment of the Stro-1^+^ population by MACS (BM after MACS) and at different passages (P0 to P3) growing under basal expansion conditions. Cells typically appeared to grow larger and were observed to display a spread morphology with increasing passage. Scale bar 500 µm (100 µm in BM) in (*a*) and 200 µm (50 µm in BM) in (*b*).

The opacity of SSCs was observed to decrease after adhesion in culture (figure 4*c*), i.e. a significant increase in membrane capacitance. At passage 0, the average opacity values were observed to fall to 0.82 ± 0.02 (*p* < 0.001, *N* = 4) with no further changes up to passage 3 when cells were kept in basal expansion conditions, which do not promote cell differentiation (figure 5*b* and electronic supplementary material, figure S4*b*). This is in keeping with significant phenotypical changes between expanded and unexpanded SSCs. Although not quantitative, optical microscopy images of SSCs following expansion indicate morphological changes such as an increase in cell size and membrane spreading with increasing passage (figure 6), which is in keeping with previous observations [16]. Higher membrane capacitance may arise from an increase in the membrane surface area and morphology, including, for example, the presence of microvilli, blebs, folds or ruffles [26–28,59], some of which may affect cell adhesion.

To assess the relationship between cell adhesion and membrane capacitance, we also analysed the dielectric properties of cancer cell lines that grow either as adherent (MG-63 and Saos-2) or as suspension (HL-60) cultures (figure 5*b*). MG-63 and Saos-2 are human osteosarcoma cell lines representative of early and mature bone cell populations, respectively, while HL-60 represents a human myeloid progenitor cell line. It is well known that cancer cells have high membrane capacitance associated with increasing structural disorder at the original tumour site and the capacity to invade neighbouring healthy tissue [28]. Higher membrane capacitance is also seen in tumour cells that originate from solid tumours. Although both MG-63 and Saos-2 cells are adherent, the membrane capacitance of MG-63 was significantly higher (*p* < 0.01) than Saos-2 (opacity values of 0.71 ± 0.03 and 0.82 ± 0.01 respectively). Conversely, although maintained under different culture methods (adherent versus suspension), Saos-2 and HL-60 showed similar opacity values with the HL-60 slightly higher (*p* < 0.05). This indicates that factors other than cell adhesion govern cells membrane capacitance. The membrane capacitance of MG-63 was also higher than expanded SSCs (*p* < 0.001, *N* = 4) confirming previous values obtained using DEP [40].

### 3.4. Cell proliferation, ALP activity and gene expression of Stro-1^+^-enriched human SSCs following cell expansion

**Figure 7.**
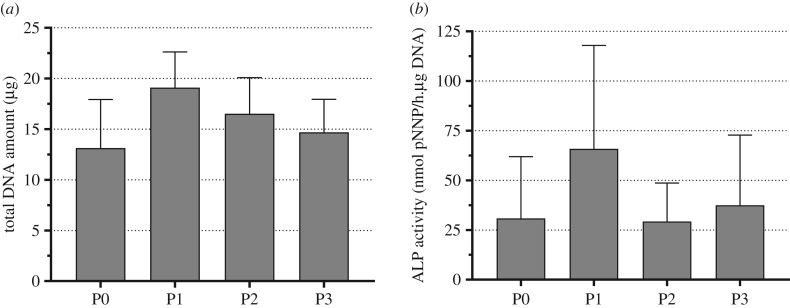
Proliferation and ALP activity of Stro-1-enriched hBMMNCs. (*a*) Total DNA quantification from hBMMNCs grown on individual six-well plates for 14 days at each passage (P0–P3) determined using the PicoGreen^®^ nucleic acid quantitation assay. (*b*) Specific ALP activity of hBMMNCs grown on individual six-well plates for 14 days at each passage determined by the colorimetric detection of the dephosphorylation of *p*-nitrophenyl phosphate. Values represent mean ± s.d. (*N* = 4).

To assess whether there were changes in the proliferation capacity or the osteogenic potential of SSCs, total DNA (figure 7*a*) and specific ALP activity were quantified at each passage (figure 7*b* and electronic supplementary material, figure S5). There were no statistical differences in the total DNA amount or ALP activity, though both were observed to be higher at passage 1. However, it is important to note that it is difficult to draw any conclusions from cell proliferation and ALP activity at passage 0, as after isolation cells are plated at a significantly higher cell density to compensate for the presence of non-adherent haematopoietic cells.

**Figure 8.**
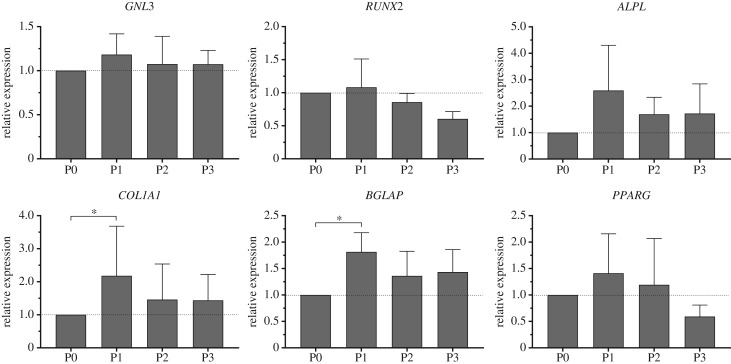
qRT-PCR. Relative gene expression of relevant genes of interest of Stro-1^+^-selected hBMMNCs grown in basal expansion conditions at different passages (P0-P3). *GNL3* is associated with stem cell proliferation, *RUNX2, ALPL, COL1A1* and *BGLAP* are genes related with the process of osteogenesis and *PPARG* is a regulator of adipocyte differentiation. Values represent mean ± s.d. normalized to the expression levels of hBMMNCs at passage 0 (*N* = 4, *p < 0.05).

The relative expression of stem and osteogenic genes of interest was assessed using quantitative reverse-transcription polymerase chain reaction (qRT-PCR) for all passages (figure 8). No differences were observed in the expression levels of *GNL3* (nucleostemin), a gene associated with stem cell proliferation. The expression of the osteogenic genes (*RUNX2, ALPL, COL1A1* and *BGLAP*) was also measured. We observed no significant differences in *ALPL* and *RUNX2*, although there was an increase in the expression of *ALPL* at passage 1 (2.6 ± 1.7-fold increase versus P0, *N* = 4) with expression levels correlating well with the specific ALP activity shown in figure 7*b*. The expression of *COL1A1* (2.2 ± 1.5-fold increase versus P0, *N* = 4) and *BGLAP* (1.8 ± 0.4-fold increase versus P0, *N* = 4) was also higher at passage 1 suggesting an enhanced osteogenic phenotype at this passage before dedifferentiation on passage. The expression levels of other genes associated with stem cell maintenance (*SOX2*), chondrogenesis (*SOX9* and *COL2A1*) and adipogenesis (*FABP2* and *PPARG*) were negligible (CT values over 30; data not shown).

### 3.5. Tracking osteogenic differentiation of Stro-1^+^-enriched human SSCs using microfluidic impedance cytometry

Microfluidic impedance cytometry and AC electrokinetic methods have demonstrated differences in the dielectric properties of stem cells and their progeny [38,42–44,60]. Exploiting these differences DEP was used to sort osteoblasts (differentiated from a human mesenchymal stem cell line for 21 days) from their progenitor cells with moderate efficiency [29]. To investigate whether impedance cytometry could be used to track osteogenic differentiation of SSCs, Stro-1^+^-enriched SSCs at passage 1 were cultured under osteogenic conditions for 14 days and the osteogenic phenotype of the cells was initially confirmed.

**Figure 9.**
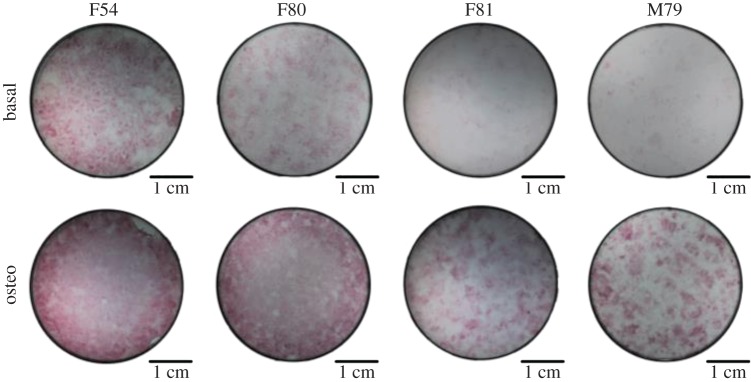
ALP activity. ALP staining of human BM colony forming units-fibroblastic (CFU-F) grown on six-well plates for 14 days under basal expansion conditions or following osteogenic (osteo) differentiation in basal medium supplemented with dexamethasone (10 nM) and ascorbate-2-phosphate (100 µM). For each individual patient (identified by genre and age), increased ALP activity can be observed following osteogenic differentiation evidenced by a stronger cell staining. Images represent an entire well captured using a dissecting stereoscope.

**Figure 10.**
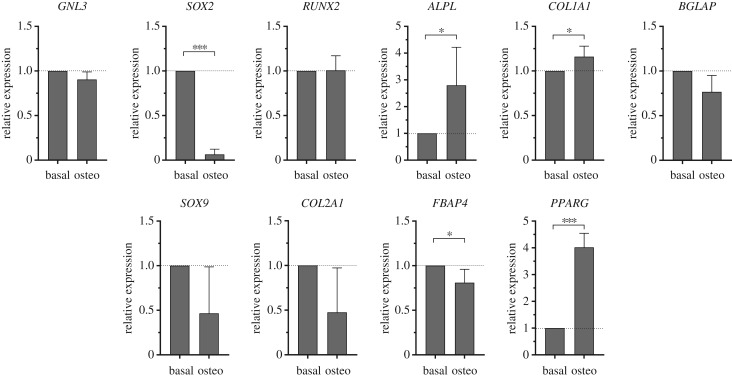
qRT-PCR. Relative gene expression of relevant genes of interest of passage 1 Stro-1^+^-selected hBMMNCs after 14 days following osteogenic (osteo) differentiation in basal medium supplemented with dexamethasone (10 nM) and ascorbate-2-phosphate (100 µM). *GNL3* and *SOX2* are associated with stem cell proliferation and stem cell maintenance, respectively. *RUNX2*, *ALPL*, *COL1A1* and *BGLAP* are genes related with the process of osteogenesis, with *RUNX2* and *BGLAP* being early and late osteogenic markers, respectively. *SOX9* and *COL2A1* are genes associated with chondrogenic differentiation, and *PPARG* and *FBAP4* with adipocyte differentiation. Values represent mean ± s.d. normalized to the expression of hBMMNCs growing under basal expansion conditions (*N* ≥ 4, *p < 0.05, ***p < 0.001).

Osteogenic induction was evidenced by an increase in ALP staining of cells grown in osteogenic conditions compared with basal expansion conditions (figure 9). This was verified by upregulation of *ALPL* (2.8 ± 1.4-fold increase, *p* < 0.05, *N* = 4) and *COL1A1* (1.2 ± 0.1-fold increase, *p* < 0.05, *N* = 4) in cells following osteogenic differentiation (figure 10). There were no differences in the expression levels of *RUNX2* and *BGLAP*, which are early and late osteogenic markers, respectively [61–63]. The expression of *GNL3*, associated with cell proliferation, was unchanged. *SOX2* was significantly downregulated (0.06 ± 0.06-fold change, *p* < 0.001, *N* = 4) although *SOX2* expression was already minimal in basal conditions. *SOX9* (0.5 ± 0.5-fold change, *N* = 4), *COL2A1* (0.5 ± 0.5-fold change, *N* = 4) and *FABP2* (0.8 ± 0.2-fold change, *N* = 4), associated with chondrogenesis and adipogenesis, respectively, were downregulated despite the high CT values at basal expansion conditions. A significant unexpected upregulation of *PPARG* (4.0 ± 0.5-fold increase, *N* = 4) in cells following osteogenic differentiation was observed, which may be due to the nature of the samples used, which typically originate from older patients undergoing total hip replacement surgery, and the increase in marrow adipogenesis associated with osteoporosis and age-related osteopenia has long been known [64,65].

Although slightly lower, the average cell size of SSCs following osteogenic differentiation (figure 5*a*, P1 Osteo, 18.5 ± 0.7 µm, *N* = 4) was not statistically distinct from SSCs following expansion (P1 Basal, 20.2 ± 0.7 µm, *N* = 4). By contrast, there was a significant increase (figure 5*b*, *p* < 0.05) in the membrane capacitance of SSCs following osteogenic differentiation reflected in a marked change in the opacity, from 0.80 ± 0.02 to 0.75 ± 0.01. Previous studies using impedance cytometry have shown a decrease in opacity (increase in membrane capacitance) of mESCs following differentiation [44]. Our results show the potential of MIC as a non-invasive and non-destructive technique to track the differentiation of SSCs without the need for any sample pre-processing. For example, using MIC it would be possible to assess the efficiency of the osteogenic differentiation of SSCs while allowing cell subculture, further biological analysis, or their use for *in vivo* implantation.

## 4. Conclusion

The biophysical properties of enriched human SSC populations (Stro-1^+^/CD146^+^) obtained from human BM change significantly under adherent expansion conditions. Specifically, this study demonstrates that both cell size and membrane capacitance of unexpanded SSCs differed from expanded cells as early as passage 0. The average cell size increased over twofold to 18.2 ± 1.3 µm while the membrane capacitance increased as determined from the decrease in electrical opacity. These results highlight the importance of studying unaltered SSCs isolated from the original tissue as the primary cell population provides a unique biophysical signature. Critically, if the size and membrane capacitance differences observed between expanded SSCs and other BM cells were verified for unexpanded SSCs, SSC isolation from human BM using microfluidic label-free sorting techniques could be easily achieved.

Nevertheless, although the membrane capacitance of the SSCs did not differ from other BM cells, the size of the SSCs in the BM was found within the largest cell fraction at 9.0 ± 0.4 µm. This is the first time that the size of the SSCs has been estimated from unexpanded human BM samples—a crucial step towards the design of a microfluidic device for SSC isolation. When compared with the size distribution of other BM cells (7.4 ± 0.2 µm and 8.3 ± 0.9 µm for Stro-1^+^/CD146^−^ and Stro-1^−^ cells, respectively), the data indicate that SSCs could be significantly enriched using a size-based sorting approach to fractionate cells smaller and larger than (e.g.) 8.5 µm, removing the vast majority of non-relevant cells in the BM. It is important to note that due to the overlapping size distribution, complete SSC isolation cannot be achieved based solely on size. One possible solution would be to design a label-free sorting system that exploits both differences in cell size with another biophysical parameter, which would lead to significant SSC enrichment. We have previously shown that SSCs at passage 0 are significantly stiffer than the three main leucocyte populations (lymphocytes, monocytes and granulocytes), also present in the BM [56]. We therefore hypothesize that cell stiffness could be used to enhance the SSC sorting purity using a sorting device that is both sensitive to size and deformation. It would be important to confirm if the higher stiffness of SSCs at passage 0 is verified in unexpanded samples, which is the subject of current investigations using deterministic lateral displacement devices (a size-based sorting technique that can provide additional data on cell deformation).

Additionally, the current study demonstrate that impedance cytometry can be used to track osteogenic differentiation of SSCs, verified by qRT-PCR of relevant genes of interest associated with stem cell maintenance and proliferation, and with the osteogenic, chondrogenic and adipogenic differentiation processes. This indicates the potential of MIC as a non-invasive and non-destructive technique to monitor stem cell differentiation in a label-free manner without the need for any sample pre-processing. The results presented in this paper will guide the design and implementation of label-free technologies for the study and isolation of primary human SSCs that could have significant impact on SSC research and clinical practice.

## Supplementary Material

Supportive supplementary material including individual patient data of fluorescence flow cytometry measurements (Fig. S1), individual scatter plots of microfluidic impedance cytometry data of human bone marrow mononuclear cells (Fig. S2), simulation data of the impedance response of cells with different membrane capacitance and radii (Fig. S3), individual measurements of cell size and opacity (Fig. S4), and alkaline phosphatase staining images from four individual patients from passage 0 to 3 (Fig. S5).

## Data accessibility

The datasets supporting this article are included in the main paper and have been uploaded as part of the electronic supplementary material. Additional datasets are available from http://doi.org/10.5258/SOTON/D0177 [66].

## References

[1] Bianco P, Robey PG 2015 Skeletal stem cells. Development 142, 1023–1027. (10.1242/dev.102210) 25758217 PMC4360182

[2] Nombela-Arrieta C, Ritz J, Silberstein LE 2011 The elusive nature and function of mesenchymal stem cells. Nat. Rev. Mol. Cell Biol. 12, 126–131. (10.1038/nrm3049) 21253000 PMC3346289

[3] Gothard D, Tare RS, Mitchell PD, Dawson JI, Oreffo ROC 2011 In search of the skeletal stem cell: isolation and separation strategies at the macro/micro scale for skeletal regeneration. Lab. Chip 11, 1206–1220. (10.1039/c0lc00575d) 21350777

[4] Aarvold A, Smith JO, Tayton ER, Jones AMH, Dawson JI, Lanham S, Briscoe A, Dunlop DG, Oreffo ROC 2014 From bench to clinic and back: skeletal stem cells and impaction bone grafting for regeneration of bone defects. J. Tissue Eng. Regen. Med. 8, 779–786. (10.1002/term.1577) 23038218

[5] Dawson JI, Kanczler J, Tare R, Kassem M, Oreffo ROC 2014 Concise review: bridging the gap: bone regeneration using skeletal stem cell-based strategies—where are we now? Stem Cells 32, 35–44. (10.1002/stem.1559) 24115290

[6] Jones E, McGonagle D 2008 Human bone marrow mesenchymal stem cells *in vivo*. Rheumatology 47, 126–131. (10.1093/rheumatology/kem206) 17986482

[7] Gothard D, Greenhough J, Ralph E, Oreffo RO 2014 Prospective isolation of human bone marrow stromal cell subsets: a comparative study between Stro-1-, CD146- and CD105-enriched populations. J. Tissue Eng. 5, 2041731414551763 (10.1177/2041731414551763) 25383172 PMC4221949

[8] Sacchetti B et al. 2007 Self-renewing osteoprogenitors in bone marrow sinusoids can organize a hematopoietic microenvironment. Cell 131, 324–326. (10.1016/j.cell.2007.08.025) 17956733

[9] Ramakrishnan A, Torok-Storb B, Pillai MM 2013 Primary marrow-derived stromal cells: isolation and manipulation. Methods Mol. Biol. 1035, 75–101. (10.1007/978-1-62703-508-8_8) 23959984 PMC3748384

[10] Simmons PJ, Torok-Storb B 1991 Identification of stromal cell precursors in human bone marrow by a novel monoclonal antibody, STRO-1. Blood 78, 55–62. 2070060

[11] Shi S, Gronthos S 2003 Perivascular niche of postnatal mesenchymal stem cells in human bone marrow and dental pulp. J. Bone Miner. Res. 18, 696–704. (10.1359/jbmr.2003.18.4.696) 12674330

[12] Fitter S, Gronthos S, Ooi SS, Zannettino ACW 2017 The mesenchymal precursor cell marker antibody STRO-1 binds to cell surface heat shock cognate 70. Stem Cells 35, 940–951. (10.1002/stem.2560) 28026090

[13] Tare RS, Babister JC, Kanczler J, Oreffo ROC 2008 Skeletal stem cells: phenotype, biology and environmental niches informing tissue regeneration. Mol. Cell. Endocrinol. 288, 11–21. (10.1016/j.mce.2008.02.017) 18395331

[14] Bianco P 2011 Bone and the hematopoietic niche: a tale of two stem cells. Blood 117, 5281–5289. (10.1182/blood-2011-01-315069) 21406722

[15] Poon Z, Lee WC, Guan G, Nyan LM, Lim CT, Han J, Van Vliet KJ 2015 Bone marrow regeneration promoted by biophysically sorted osteoprogenitors from mesenchymal stromal cells. Stem Cells Transl. Med. 4, 56–65. (10.5966/sctm.2014-0154) 25411477 PMC4275011

[16] Whitfield MJ, Lee WCJ, Van Vliet KJ 2013 Onset of heterogeneity in culture-expanded bone marrow stromal cells. Stem Cell Res. 11, 1365–1377. (10.1016/j.scr.2013.09.004) 24103495

[17] Maloney JM, Nikova D, Lautenschläger F, Clarke E, Langer R, Guck J, Van Vliet KJ 2010 Mesenchymal stem cell mechanics from the attached to the suspended state. Biophys. J. 99, 2479–2487. (10.1016/j.bpj.2010.08.052) 20959088 PMC2955350

[18] Bruder SP, Jaiswal N, Haynesworth SE 1997 Growth kinetics, self-renewal, and the osteogenic potential of purified human mesenchymal stem cells during extensive subcultivation and following cryopreservation. J. Cell. Biochem. 64, 278–294. 10.1002/(SICI)1097-4644(199702)64:2%3C278::AID-JCB11%3E3.0.CO;2-F) 9027588

[19] Banfi A, Muraglia A, Dozin B, Mastrogiacomo M, Cancedda R, Quarto R 2000 Proliferation kinetics and differentiation potential of *ex vivo* expanded human bone marrow stromal cells: Implications for their use in cell therapy. Exp. Hematol. 28, 707–715. (10.1016/S0301-472X(00)00160-0) 10880757

[20] Bonab MM, Alimoghaddam K, Talebian F, Ghaffari SH, Ghavamzadeh A, Nikbin B 2006 Aging of mesenchymal stem cell *in vitro*. BMC Cell Biol. 7, 14 (10.1186/1471-2121-7-14) 16529651 PMC1435883

[21] Kim J et al. 2009 Biological characterization of long-term cultured human mesenchymal stem cells. Arch. Pharm. Res. 32, 117–126. (10.1007/s12272-009-1125-1) 19183884

[22] Gothard D, Cheung K, Kanczler JM, Wilson DI, Oreffo ROC 2015 Regionally-derived cell populations and skeletal stem cells from human foetal femora exhibit specific osteochondral and multi-lineage differentiation capacity *in vitro* and *ex vivo*. Stem Cell Res. Ther. 6, 251 (10.1186/s13287-015-0247-2) 26684339 PMC4683700

[23] Stewart K, Walsh S, Screen J, Jefferiss CM, Chainey J, Jordan GR, Beresford JN 1999 Further characterization of cells expressing STRO-1 in cultures of adult human bone marrow stromal cells. J. Bone Miner. Res. 14, 1345–1356. (10.1359/jbmr.1999.14.8.1345) 10457267

[24] Halfon S, Abramov N, Grinblat B, Ginis I 2011 Markers distinguishing mesenchymal stem cells from fibroblasts are downregulated with passaging. Stem Cells Dev. 20, 53–66. (10.1089/scd.2010.0040) 20528146

[25] Xavier M, Oreffo ROC, Morgan H 2016 Skeletal stem cell isolation: a review on the state-of-the-art microfluidic label-free sorting techniques. Biotechnol. Adv. 34, 908–923. (10.1016/j.biotechadv.2016.05.008) 27236022

[26] Pethig R 2013 Review Article—dielectrophoresis: status of the theory, technology, and applications review article—dielectrophoresis : status of the theory, technology, and applications. Biomicrofluidics 4, 22811 (10.1063/1.3456626) PMC2917862 20697589

[27] Pethig R, Menachery A, Pells S, De Sousa P 2010 Dielectrophoresis: a review of applications for stem cell research. J. Biomed. Biotechnol. 2010, 1–7. (10.1155/2010/182581) PMC2871555 20490279

[28] Gascoyne PRC, Shim S 2014 Isolation of circulating tumor cells by dielectrophoresis. Cancers 6, 545–579. (10.3390/cancers6010545) 24662940 PMC3980488

[29] Song H et al. 2015 Continuous-flow sorting of stem cells and differentiation products based on dielectrophoresis. Lab. Chip 15, 1320–1328. (10.1039/C4LC01253D) 25589423 PMC8385543

[30] Muratore M, Srsen V, Waterfall M, Downes A, Pethig R 2012 Biomarker-free dielectrophoretic sorting of differentiating myoblast multipotent progenitor cells and their membrane analysis by Raman spectroscopy. Biomicrofluidics 6, 034113 (10.1063/1.4746252) PMC3432085 23940503

[31] Nourse JL, Prieto JL, Dickson AR, Lu J, Pathak MM, Tombola F, Demetriou M, Lee AP, Flanagan LA 2014 Membrane biophysics define neuron and astrocyte progenitors in the neural lineage. Stem Cells 32, 706–716. (10.1002/stem.1535) 24105912 PMC5367858

[32] Vykoukal J, Vykoukal DM, Freyberg S, Alt U, Gascoyne PRC 2008 Enrichment of putative stem cells from adipose tissue using dielectrophoretic field-flow fractionation. Lab. Chip 8, 1386–1393. (10.1039/b717043b) 18651083 PMC2726253

[33] Stephens M, Talary MS, Pethig R, Burnett AK, Mills KI 1996 The dielectrophoresis enrichment of CD34^+^ cells from peripheral blood stem cell harvests. Bone Marrow Transplant. 18, 777–782. 8899194

[34] Talary MS, Mills KI, Hoy T, Burnett AK, Pethig R 1995 Dielectrophoretic separation and enrichment of CD34^+^ cell subpopulation from bone marrow and peripheral blood stem cells. Med. Biol. Eng. Comput. 33, 235–237. (10.1007/BF02523050) 7543968

[35] Su HW, Prieto JL, Voldman J 2013 Rapid dielectrophoretic characterization of single cells using the dielectrophoretic spring. Lab. Chip 13, 4109–4117. (10.1039/c3lc50392e) 23970334

[36] Pethig R, Talary MS 2007 Dielectrophoretic detection of membrane morphology changes in Jurkat T-cells undergoing etoposide-induced apoptosis. IET Nanobiotechnol. 1, 2 (10.1049/iet-nbt:20060018) 17500582

[37] Velugotla S, Pells S, Mjoseng HK, Duffy CRE, Smith S, De Sousa P Pethig R 2012 Dielectrophoresis based discrimination of human embryonic stem cells from differentiating derivatives. Biomicrofluidics 6, 44113 (10.1063/1.4771316) 24339846 PMC3555604

[38] Flanagan LA, Lu J, Wang L, Marchenko SA, Jeon NL, Lee AP, Monuki ES 2008 Unique dielectric properties distinguish stem cells and their differentiated progeny. Stem Cells 26, 656–665. (10.1634/stemcells.2007-0810) 18096719

[39] Labeed FH, Lu J, Mulhall HJ, Marchenko SA, Hoettges KF, Estrada LC, Lee AP, Hughes MP, Flanagan LA 2011 Biophysical characteristics reveal neural stem cell differentiation potential. PLoS ONE 6, 1–11. (10.1371/journal.pone.0025458) PMC3184132 21980464

[40] Ismail A, Hughes M, Mulhall H, Oreffo R, Labeed F 2015 Characterization of human skeletal stem and bone cell populations using dielectrophoresis. J. Tissue Eng. Regen. Med. 9, 162–168. (10.1002/term.1629) 23225773

[41] Sun T, Morgan H 2010 Single-cell microfluidic Impedance cytometry: a review. Microfluid Nanofluidics 8, 423–443. (10.1007/s10404-010-0580-9)

[42] Zhao Y et al. 2016 Electrical property characterization of neural stem cells in differentiation. PLoS ONE 11, e0158044 (10.1371/journal.pone.0158044) 27341032 PMC4920408

[43] Song H, Wang Y, Rosano JM, Prabhakarpandian B, Garson C, Pant K, Lai E 2013 A microfluidic impedance flow cytometer for identification of differentiation state of stem cells. Lab. Chip 13, 2300–2310. (10.1039/c3lc41321g) 23636706

[44] Zhou Y, Basu S, Laue E, Seshia AA 2016 Single cell studies of mouse embryonic stem cell (mESC) differentiation by electrical impedance measurements in a microfluidic device. Biosens. Bioelectron. 81, 249–258. (10.1016/j.bios.2016.02.069) 26963790 PMC4833703

[45] Tare RS, Mitchell PD, Kanczler J, Oreffo ROC 2012 Bone research protocols. New York, NY: Springer.

[46] Williams EL, White K, Oreffo ROC 2013 Isolation and enrichment of stro-1 immunoselected mesenchymal stem cells from adult human bone marrow. Methods Mol. Biol. 1035, 67–73. (10.1007/978-1-62703-508-8_7) 23959983

[47] Dawson JI, Smith JO, Aarvold A, Ridgway JN, Curran SJ, Dunlop DG, Oreffo ROC 2013 Enhancing the osteogenic efficacy of human bone marrow aspirate: concentrating osteoprogenitors using wave-assisted filtration. Cytotherapy 15, 242–252. (10.1016/j.jcyt.2012.09.004) 23245952

[48] Holmes D, She JK, Roach PL, Morgan H 2007 Bead-based immunoassays using a micro-chip flow cytometer. Lab. Chip 7, 1048–1056. (10.1039/b707507n) 17653348

[49] Morgan H, Holmes D, Green NG 2006 High speed simultaneous single particle impedance and fluorescence analysis on a chip. Curr. Appl. Phys. 6, 367–370. (10.1016/j.cap.2005.11.020)

[50] Spencer D, Hollis V, Morgan H 2014 Microfluidic impedance cytometry of tumour cells in blood. Biomicrofluidics 8, 064124 (10.1063/1.4904405) 25553198 PMC4265026

[51] de Andrés MC, Imagawa K, Hashimoto K, Gonzalez A, Roach HI, Goldring MB, Oreffo ROC 2013 Loss of methylation in CpG sites in the NF-κB enhancer elements of inducible nitric oxide synthase is responsible for gene induction in human articular chondrocytes. Arthritis Rheum. 65, 732–742. (10.1002/art.37806) 23239081 PMC3937961

[52] Janeczek AA, Tare RS, Scarpa E, Moreno-Jimenez I, Rowland CA, Jenner D, Newman TA, Oreffo ROC, Evans ND 2016 Transient canonical wnt stimulation enriches human bone marrow mononuclear cell isolates for osteoprogenitors. Stem Cells 34, 418–430. (10.1002/stem.2241) 26573091 PMC4981914

[53] Janeczek AA 2015 Wnt protein delivery to skeletal stem cells for bone tissue regeneration. Southampton, UK: University of Southampton.

[54] Lee WC et al. 2014 Multivariate biophysical markers predictive of mesenchymal stromal cell multipotency. Proc. Natl Acad. Sci. USA 111, E4409–E4418. (10.1073/pnas.1402306111) 25298531 PMC4210311

[55] Xavier M et al. 2016 Mechanical phenotyping of primary human skeletal stem cells in heterogeneous populations by real-time deformability cytometry. Integr. Biol. 8, 616–623. (10.1039/C5IB00304K) 26980074

[56] Lo Surdo J, Bauer SR 2012 Quantitative approaches to detect donor and passage differences in adipogenic potential and clonogenicity in human bone marrow-derived mesenchymal stem cells. Tissue Eng. Part C Methods 18, 877–889. (10.1089/ten.tec.2011.0736) 22563812 PMC3483050

[57] Hur SC, Henderson-MacLennan NK, McCabe ERB, Di Carlo D 2011 Deformability-based cell classification and enrichment using inertial microfluidics. Lab. Chip 11, 912–920. (10.1039/c0lc00595a) 21271000

[58] Holmes D, Whyte G, Bailey J, Vergara-Irigaray N, Ekpenyong A, Guck J, Duke T 2014 Separation of blood cells with differing deformability using deterministic lateral displacement. Interface Focus 4, 1–10. (10.1098/rsfs.2014.0011) PMC4213443 25485078

[59] Gascoyne PRC, Shim S, Noshari J, Becker FF, Stemke-Hale K 2013 Correlations between the dielectric properties and exterior morphology of cells revealed by dielectrophoretic field-flow fractionation. Electrophoresis 34, 1042–1050. (10.1002/elps.201200496) 23172680 PMC3754903

[60] Bagnaninchi PO, Drummond N 2011 Real-time label-free monitoring of adipose-derived stem cell differentiation with electric cell-substrate impedance sensing. Proc. Natl Acad. Sci. USA 108, 6462–6467. (10.1073/pnas.1018260108) 21464296 PMC3080969

[61] Bruderer M, Richards R, Alini M, Stoddart M 2014 Role and regulation of RUNX2 in osteogenesis. Eur. Cells Mater. 28, 269–286. (10.22203/eCM.v028a19) 25340806

[62] Granéli C, Thorfve A, Ruetschi U, Brisby H, Thomsen P, Lindahl A, Karlsson C 2014 Novel markers of osteogenic and adipogenic differentiation of human bone marrow stromal cells identified using a quantitative proteomics approach. Stem Cell Res. 12, 153–165. (10.1016/j.scr.2013.09.009) 24239963

[63] Baek W-Y, Lee M-A, Jung JW, Kim S-Y, Akiyama H, de Crombrugghe B, Kim J-E 2009 Positive regulation of adult bone formation by osteoblast-specific transcription factor osterix. J. Bone Miner. Res. 24, 1055–1065. (10.1359/jbmr.081248) 19113927 PMC4020416

[64] Nuttall M, Gimble J 2004 Controlling the balance between osteoblastogenesis and adipogenesis and the consequent therapeutic implications. Curr. Opin. Pharmacol. 4, 290–294. (10.1016/j.coph.2004.03.002) 15140422

[65] Akune T et al. 2004 PPARgamma insufficiency enhances osteogenesis through osteoblast formation from bone marrow progenitors. J. Clin. Invest. 113, 846–855. (10.1172/JCI200419900) 15067317 PMC362117

[66] Xavier M, de Andrés MC, Spencer D, Oreffo ROC, Morgan H 2017 Data from: Size and dielectric properties of skeletal stem cells change critically after enrichment and expansion from human bone marrow: consequences for microfluidic cell sorting. *Dryad Digital Repository*. (10.5258/SOTON/D0177) PMC5582119 28835540

