# Supplementary material for: Size and dielectric properties of skeletal stem cells change critically after enrichment and expansion from human bone marrow: consequences for microfluidic cell sorting
Source: J R Soc Interface. 2017 Aug 23;14(133):20170233. doi: 10.1098/rsif.2017.0233 (PMC5582119; doi:10.1098/rsif.2017.0233)
Supplement: Supportive supplementary material including individual patient data of fluorescence flow cytometry measurements (Fig. S1), individual scatter plots of microfluidic impedance cytometry data of human bone marrow mononuclear cells (Fig. S2), simulation data of the impedance response of cells with diffe [file rsif20170233supp1.pdf]

Size and Dielectric Properties of Skeletal Stem Cells Change Critically After Enrichment and Expansion from Human Bone Marrow: Consequences for Microfluidic Cell Sorting

Miguel Xavier [a,b], María C. de Andrés [b], Daniel Spencer [a], Richard OC Oreffo [b*] and Hywel Morgan [a*]

[a] Faculty of Physical Sciences and Engineering, and Institute for Life Sciences, University of Southampton, SO17 1BJ, United Kingdom.

[b] Centre for Human Development, Stem Cells and Regeneration, Institute of Developmental Sciences, Southampton General Hospital, Tremona Road, SO16 6YD Southampton, United Kingdom.

[*] Joint corresponding authors

Electronic Supplementary Material

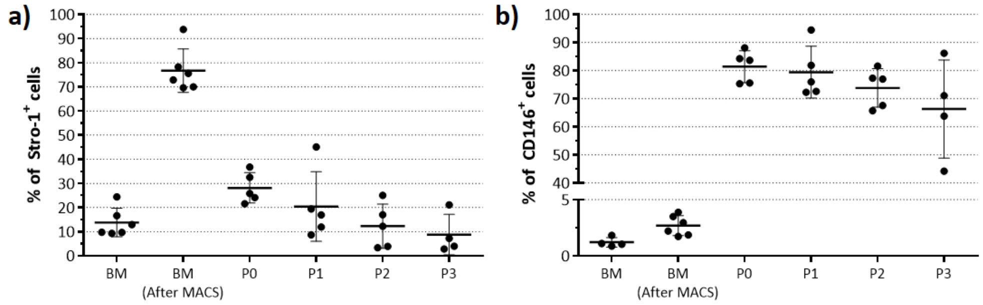

Figure S1. Fluorescence flow cytometry of human bone marrow mononuclear cells (hBMMNCs). Frequency of Stro-1$^+$ (a) and CD146$^+$ (b) hBMMNCs in the BM, before and after enrichment of the Stro-1$^+$ population by MACS, and following cell expansion under basal culture conditions (passages 0 to 3). Values represent mean ± SD for each individual analysed patient sample.

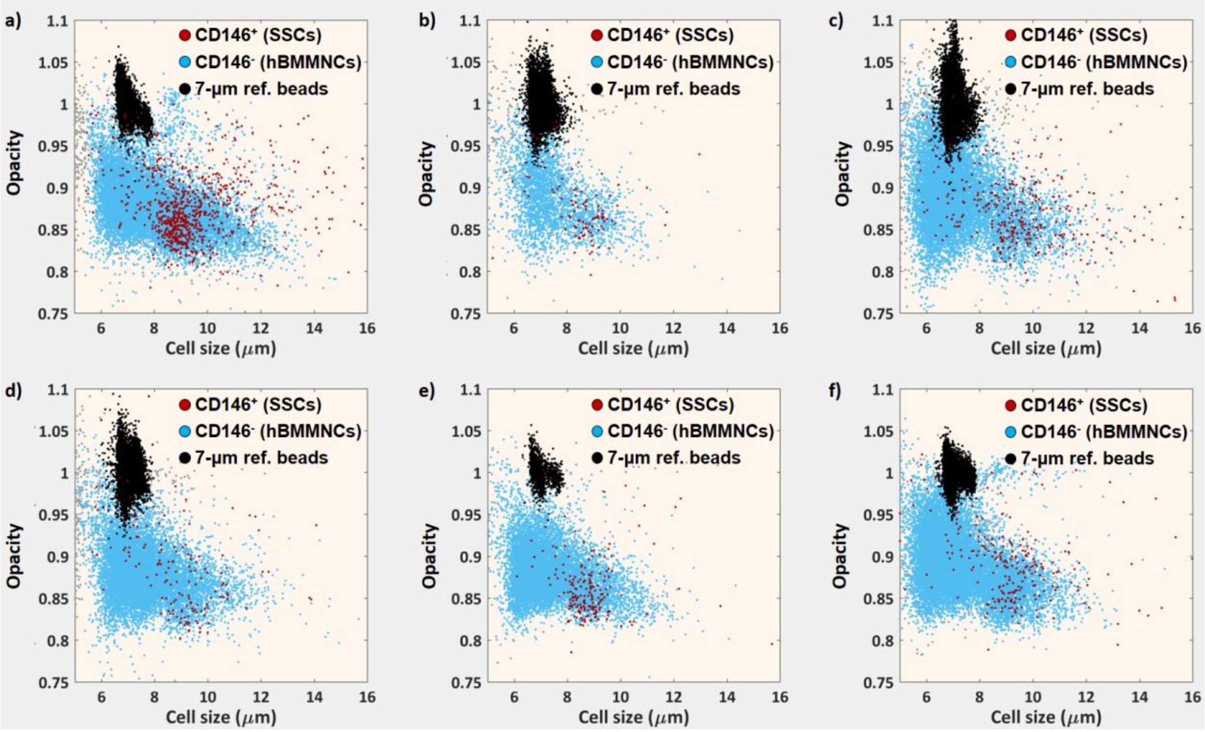

Figure S2. Fluorescence-assisted microfluidic impedance cytometry of Stro-1-enriched human bone marrow mononuclear cells (hBMMNCs). a-f) Scatter plots of cell size (µm) *vs* electrical opacity (normalised to 7-µm diameter reference beads) of Stro-1$^+$-enriched hBMMNCs obtained from six individual patients.

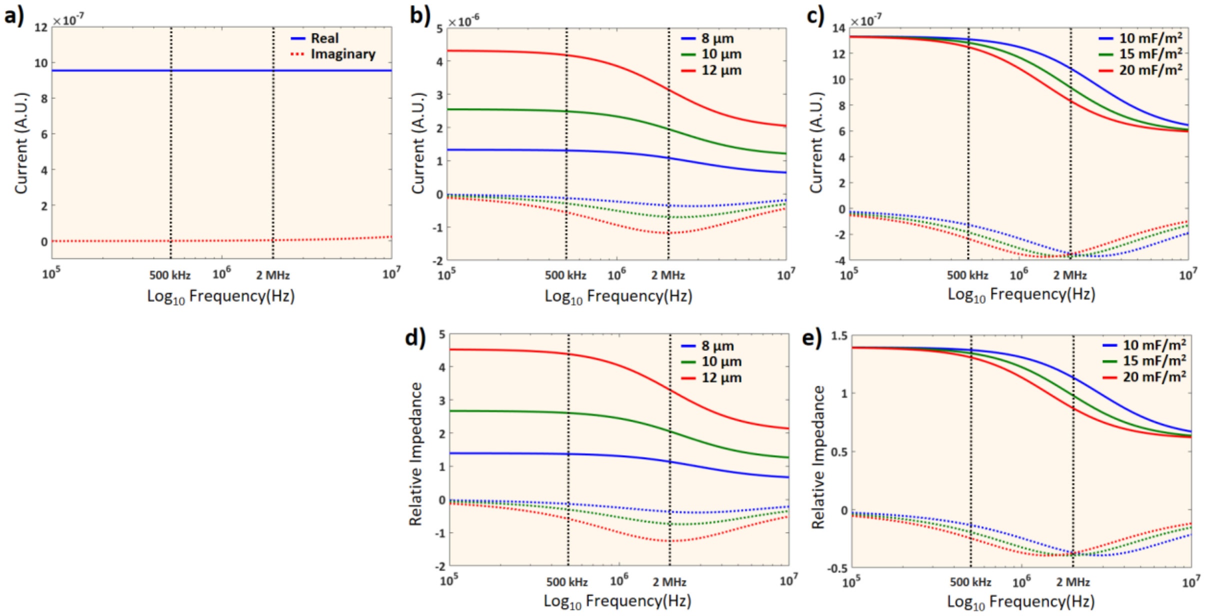

Figure S3. Simulation (in MATLAB™) of the impedance response of the 7-µm polystyrene reference beads (a) and cells (b-c) using a simplified single-shell model with a suspending medium of conductivity 1.6 S/m. The data shows three different cells, which differ in size (8 to 12 µm), or membrane capacitance (10 to 20 mF/m^2). d) and e) show the impedance response of the cells normalised to the reference beads. The two frequencies used in the study to calculate cell size (500 kHz) and opacity (2 MHz) are indicated by vertical dashed lines. Coloured full and dashed lines represent the real and imaginary parts of the signal respectively. The following parameters were used: membrane thickness, 5 nm, medium relative permittivity, 78.5, membrane conductivity, 10^{-5} S/m, cytoplasm conductivity, 0.8 S/m and cytoplasm relative permittivity, 60.

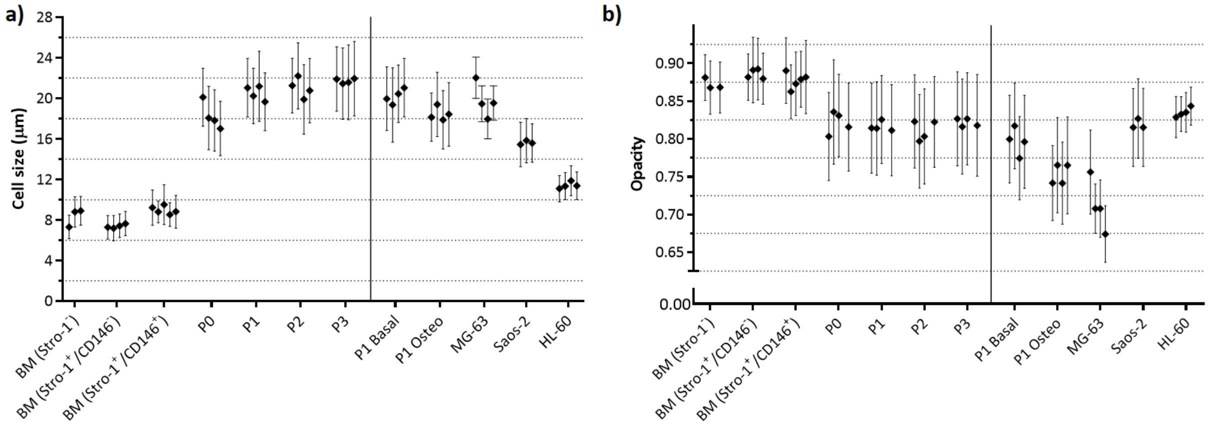

Figure S4. Size and electrical opacity of cells analysed by microfluidic impedance cytometry. Cell size (a) and electrical opacity (b) of hBMMNCs in the bone marrow (BM), after expansion at passages 0 to 3, and following osteogenic differentiation (P1 Osteo). Within the BM, cell size and opacity were measured for Stro-1$^-$ cells, and both the CD146$^-$ and CD146$^+$ populations within the Stro-1$^+$ fraction. Skeletal stem cells (SSCs) are contained within the Stro-1$^+$/CD146$^+$ population. Also shown are data for two adherent (MG-63 and Saos-2) and one suspension (HL-60) human cancer cell lines. Values represent mean ± SD for each individual analysed patient sample.

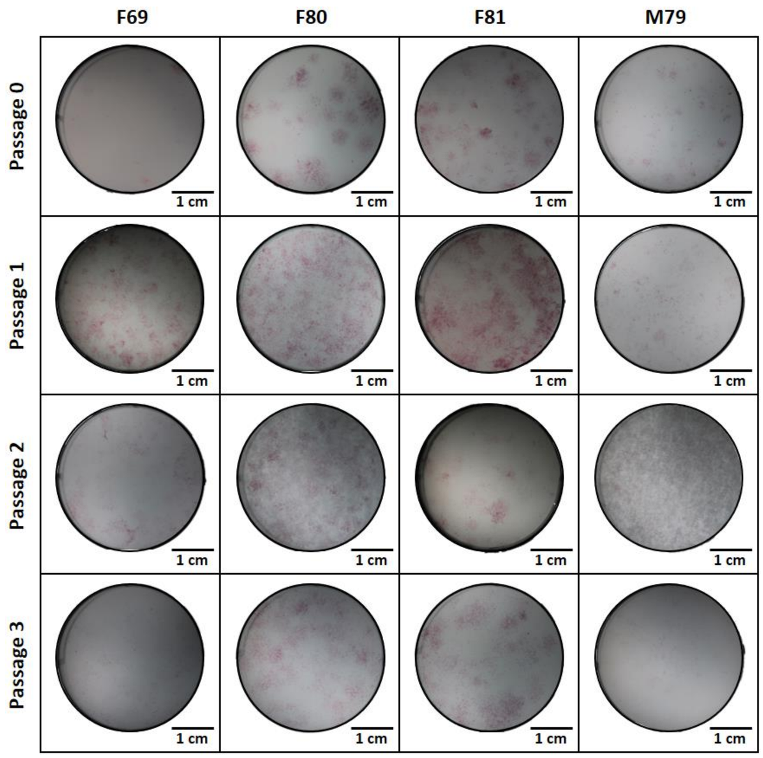

Figure S5. Alkaline phosphatase activity. Alkaline phosphatase staining of human BM CFU-F colonies grown under basal expansion conditions at different passages (passage 0 to passage 3).
